# Proceedings of the Hahnemannian Association of Penna.

**Published:** 1888-11

**Authors:** 


### PROCEEDINGS OF THE HAHNEMANNIAN ASSOCIATION OF PENNSYLVANIA.

The stated meeting of the "Hahnemannian Association of Pennsylvania" was held in the Continental Hotel, Philadelphia, Tuesday evening, September 13th. After routine business the following interesting paper from Dr. E. W. Berridge (honorary member) was read, causing a spirited and instructive discussion.

